# Variants of the Progesterone Receptor Gene as Modulators of Risk for Idiopathic Spontaneous Premature Birth

**DOI:** 10.3390/ijms26041606

**Published:** 2025-02-13

**Authors:** Mirta Kadivnik, Marija Dundović, Andreja Bartulić, Vinka Rupčić Rubin, Kristina Abičić Žuljević, Iva Milić Vranješ, Kristina Kralik, Nena Arvaj, Jasenka Wagner

**Affiliations:** 1 Clinic of Obstetrics and Gynecology, University Hospital Center Osijek, J. Huttlera 4, 31000 Osijek, Croatia; mirta.kadivnik@kbco.hr (M.K.);; 2 Department of Obstetrics and Gynecology, Faculty of Medicine, J.J. Strossmayer University, 31000 Osijek, Croatia; 3 Department of Internal Medicine, University Hospital Center Osijek, 31000 Osijek, Croatia; 4 Department of Internal Medicine, Faculty of Medicine, J.J. Strossmayer University, 31000 Osijek, Croatia; 5 Department of Medical Statistics and Informatics, Faculty of Medicine, J.J. Strossmayer University, 31000 Osijek, Croatia; 6 Department of Medical Biology and Genetics, Faculty of Medicine, J.J. Strossmayer University, 31000 Osijek, Croatia

**Keywords:** genetic variation, premature birth, progesterone, progesterone receptors, single-nucleotide polymorphisms

## Abstract

Premature birth (PTB) is the most common cause of perinatal mortality and morbidity. We performed a case–control study to determine whether two selected single-nucleotide polymorphisms (SNPs) of the progesterone receptor gene (*PGR*) (rs4754732 and rs653752) play a role in the modulation of the risk for spontaneous PTB. This study included 400 mothers (199 with premature delivery and 201 with term delivery) and 400 newborns (201 term-born and 199 premature-born) of European descent. Genotyping was performed with an ABI PRISM 7500 SDS using TaqMan SNP genotyping assays. We found no statistically significant difference in the distribution of genotypes and allele frequencies between prematurely born newborns and newborns at term for either investigated SNP. There was no statistically significant difference in the distribution of genotypes and allele frequencies between groups of mothers with extremely early and early PTB compared to the group of mothers with term births. Potential association of the mothers’ C allele of rs653752 with lower odds of PTB (*p* = 0.03; odds ratio 1.36; 95% confidence interval 1.02–1.81; Chi-square test), and association of the mothers’ CC genotype of rs653752 in the recessive inheritance model with lower odds of PTB in general (*p* = 0.02; odds ratio 0.54; 95% confidence interval 0.32–0.91; Chi-square test) and with a late PTB (*p* = 0.005, odds ratio 0.45, 95% confidence interval 0.23–0.79; Chi-square test), were found. It was also found that the mothers who were carriers of the haplotype T-G combination of rs4754732 and rs653752 were 1.5 times more likely to have PTB, even after correcting the *p*-value for multiple comparisons (*p* = 0.008; odds ratio 1.59; 95% confidence interval 1.13–2.24, Chi-square test). Further research on a larger number of subjects of these and other *PGR* SNPs will be needed in order to confirm the presented results.

## 1. Introduction

Premature birth (PTB) is defined by the World Health Organization (WHO) as a live birth before 37 weeks of gestation [1,2]. In 2020, the percentage of PTBs worldwide was 9.9%, ranging from around 6.8% in East Asia, Southeast Asia, and Oceania to 13.2% in South Asia (as high as 16.2% in Bangladesh) [3]. In the Republic of Croatia, the percentage of PTBs varied between 6.19% and 6.97% between 1994 and 2014 [4]. PTB is associated with 70% of neonatal mortalities and 75% of neonatal morbidities [5].

Progesterone (P4) is a critical hormone involved with pregnancy maintenance; its absence or relative absence is associated with pregnancy failure, preterm labor, and other poor outcomes [6]. It establishes and maintains pregnancy by inhibiting cervical maturation [7] and reducing the expression of inflammatory cytokines/chemokines (e.g., interleukin-1 and interleukin-8) [8]. It also prevents apoptosis in the membranes of the fetal amnion under both normal and proinflammatory conditions and prevents membrane rupture and PTB [9,10]. P4 has been the focus of several recent investigations of therapeutic modalities for PTB [11,12]. Additional studies have examined other P4 formulations in various high-risk cohorts and have also shown therapeutic benefits with P4 [13].

The physiological effects of P4 are mediated by binding to specific nuclear progesterone receptors (nPRs). They act by modulating the expression of specific downstream target genes such as the Gap junction Protein Alpha 1 (*GJA1*), the Prostaglandin-Endoperoxide Synthase 2 gene (*PTGS2*), the oxytocin receptor gene (*OXTR*), and the nuclear factor kappa-light-chain-enhancer of activated B-cells subunit (*NF-κB2*) [14,15]. Two isoforms of PRs are crucial for the influence of P4 on the onset of labor: PR-A and PR-B. Progesterone receptor-A (PR-A) is smaller, lacks the 164 N-terminal amino acids that form an activation domain 3 on the receptor, and is thought to inhibit the transcription of progesterone-responsive genes. It also acts by increasing the expression of the proinflammatory genes’ interleukin 1A and interleukin 8 [16]. In contrast, progesterone receptor-B (PR-B) increases the transcription of progesterone-responsive genes and has an overall quiescent effect on the myometrium. It also inhibits the transcription of proinflammatory genes [17,18].

One recognized risk factor for PTB is maternal and/or fetal genetic predisposition. This has been confirmed in many epidemiological studies [19,20,21]. Studies suggest that maternal genetic variants contribute about 20.6 to 25% to the heritability of PTB [21,22].

The progesterone receptor gene (*PGR*) codes progesterone receptors (PRs). The human *PGR* is located on the long arm of chromosome 11 (cytogenetic band 11q21.1), and it consists of eight exons and seven introns [23].

Many single-nucleotide polymorphisms (SNPs) have been described in human *PGR.* Variations in deoxyribonucleic acid (DNA) nucleotides’ sequences may contribute to different susceptibility to disease and individual responses to treatment or environmental factors [24,25]. SNPs in the coding and noncoding regions of genes cause other changes. An SNP in the coding region of a gene has pathogenic impacts on the protein structure, function, stability, and solubility through amino acid replacement in the protein sequence [26]. In contrast, an SNP in a noncoding region potentially affects the gene expression [27,28].

Previous studies have included several variants of maternal and/or newborn *PGR* (Ehn et al. had the most in their research, with 18 in total [29]), and the results of the PTB risk modulation have so far been contradictory [10,12,29,30,31,32,33]. It is assumed that changes in *PGR*s influence the change in the ratio of intracellular PRs, the sensitivity of PRs to the effect of P4, and further P4/PR signaling, as well as the expression of the *PGR*s themselves [10,34].

Based on the results of previous studies, we selected two SNPs in maternal and newborn *PGR* that might be associated with a higher risk of a PTB (rs4754732 and rs653752) [12,29,31,32].

rs4754732 (11_101008502_T>A/T>C; minor allele frequency (MAF) C 0.313) is a variant of *PGR* which is located in the promoter region of *PGR* and has a role in the alteration in the genetic expression of PR isoforms [29,35].

rs653752 (11_101077379_C>G; MAF C 0.356) is located in the regulatory region of *PGR,* and its impact on PR is considered to also be in terms of the regulation of *PGR* gene expression [29].

Our previous pilot case–control study included research on four SNPs of *PGR* (rs1042838, rs1042839, rs1942836, and rs10895068), and three of them in mothers and newborns (rs1042838, rs1042839, and rs1942836) showed that they might be associated with a higher PTB risk [36].

The aim of this study was to investigate the role of the other two selected genetic variations mentioned above in neonatal and maternal *PGR* (rs4754732 and rs653752) and to identify women at higher or lower risk for PTB compared to the general population. These genetic markers can also be used for risk stratification of pregnancies. In addition, the identification of genetic variants of *PGR* that are associated with a high risk of preterm birth could lead to the targeted use of progesterone intervention therapies in the future.

## 2. Results

For this case–control study, 400 pregnant mothers and 400 newborns were included. The mothers were stratified with regard to gestational age at birth as either term (n = 201) or preterm (n = 199), while the newborns were stratified with regard to time of birth as either term (n = 201) or preterm (n = 199). Table 1 shows the demographic characteristics of the mothers and newborns in the PTB and control groups and selected risk factors for preterm birth.

In the total number of PTBs, the rate of extremely early PTBs was 8%, the rate of early PTBs was 17%, and the rate of late PTBs was 75%. These data follow the world literature data [37].

The genotype frequencies of the investigated polymorphisms in the study were in Hardy–Weinberg equilibrium across all groups (*p* > 0.05) (Tables S1–S10).

There was a statistically significant difference between the groups of mothers with a term birth and a PTB in the frequency of the mothers’ C allele of rs653752 (odds ratio (OR) 1.36, 95% confidence interval (CI) 1.02–1.81, *p* = 0.03, Chi-square test) (Table 2). Mothers with the C/C genotype in the recessive inheritance model (CC vs. GG + CG) had a less than two times lower chance of a PTB (OR 0.54, CI 95% 0.32–0.91, *p* = 0.03) (Table 3). There was no statistically significant difference in the frequency of alleles and distribution of genotypes between the groups of prematurely born newborns and newborns born at term (Tables S1 and S2, Supplementary Materials).

After primary analysis, we divided the group of mothers with a PTB and prematurely born newborns into three subgroups: extremely early PTB (22 to 27 + 6 weeks of gestation), early PTB (28 to 31 + 6 weeks of gestation), and late PTB (32 to 36 + 6 weeks of gestation). We compared each of these subgroups of mothers and newborns separately with the group of mothers and newborns with term birth.

When we compared the groups of mothers with a late PTB and mothers with a term birth, we found a statistically significant difference between the cases and controls in the frequency of the mothers’ C allele of rs653752 (OR 1.44, 95% CI 1.06–1.96, *p* = 0.02, Chi-square test) in favor of a term birth and the CC genotype distribution of rs653752 also in favor of a term birth (OR 0.42, 95% CI 0.21–0.81, Chi-square test) (Table 4). Mothers with the C/C genotype in the recessive inheritance model (CC vs. GG + CG) had a slightly more than two times lower chance for a late PTB (OR 0.43, CI 95% 0.23–0.79, *p* = 0.005) (Table 5).

There was no statistically significant difference in the distribution of genotypes and frequencies of alleles between the groups of mothers with an extremely early PTB and an early PTB compared to the group of mothers with a term birth (Tables S3 and S4, Supplementary Materials). In addition, we found no statistically significant difference in the distribution of genotypes and frequencies of alleles between the groups of premature newborns who were born extremely early, early, or late compared to the group of newborns born at term (Tables S5–S10, Supplementary Materials).

When analyzing the haplotypes, we found just one haplotype of these two SNPs of *PGR* in mothers associated with a PTB. The mothers who were carriers of the haplotype T G combination of SNPs rs4754732 and rs653752 had a 1.5 times higher chance of having a PTB even after correction of the *p*-value for multiple comparisons (OR 1.59, CI 95% 1.13–2.24, *p* = 0.008) (Table 6).

Next, we conducted bivariate logistic regression to predict the PTB probability associated with the two SNPs of *PGR* in the newborns and mothers. There was no statistically significant model for PTB prediction (Table 7).

## 3. Discussion

Despite numerous studies on the mechanisms of its development and the preventive and therapeutic measures that could and should influence its incidence, PTB is still one of the main causes of high rates of perinatal mortality and morbidity [38]. Among all the etiological factors that cause PTB, the genetic predisposition to PTB is increasingly gaining attention. It has been shown that there is an increased risk of PTB in mothers who either had a PTB themselves or have a family history of PTBs [22,39,40].

Many genes and SNPs and their association with a PTB have been investigated to explore the pathophysiological pathways that subsequently lead to a PTB [41,42]. In addition to the maternal inflammatory response as one of the main mechanisms leading to a PTB, it is certainly worth noting the effects of P4 on pregnancy maintenance and possible alterations in signaling that could lead to a PTB. It is known that, in humans, in contrast to other mammals, there is no classic drop in the P4 concentration in blood plasma, which leads to birth, among other things. In humans, however, we know of the functional progesterone withdrawal (FPW) phenomenon, which is caused by a change in the function or expression of PR to which P4 is bound. Therefore, this process could be one of the triggers for a PTB [43,44,45].

In the spirit of the previous statements, we started an investigation on various SNPs of *PGR* and their association with PTB, assuming that certain polymorphisms of a single *PGR* nucleotide are at least partially responsible for the increased odds ratio for a PTB.

In our first pilot study, we analyzed four SNPs of *PGR*, and we showed that three of them (rs1942836, rs1042838, and rs1042839) have an association with the occurrence of a PTB either in mothers or in newborns [36]. In this study, we analyzed two additional SNPs of *PGR* that also have an impact on *PGR* expression and the regulation of PR-A and PR-B, as well as further P4/PR signaling (rs4754732 and rs653752).

Our results showed that mothers who are carriers of the homozygous CC genotype of rs653752 in a recessive inheritance model were almost twice as likely to have a term birth compared to a group of mothers with PTB and, subsequently, a group of mothers with late PTB. These results suggest a potential protective role of the CC genotype in pregnancy maintenance, likely mediated through *PGR* function.

In contrast to some other SNPs of *PGR*, the SNP rs653752 has been studied much less in connection with PTB. It is an intron variant that is located in the regulatory region of *PGR* and presumably influences the expression of *PGR.* To date, four studies have been conducted to examine the association of the rs653752 with a higher or lower incidence of PTB. Only Ehn et al. found a significant association of the abovementioned SNP of *PGR* with PTB. Namely, Ehn et al. found the association of the mentioned SNP of *PGR* in mothers with an early PTB and with PTB in general [29]. In the study by Bustos et al. [32], this SNP was not in HW equilibrium and was excluded from further analysis, while in the other two studies, Mann et al. [31] and Manuck et al. [12], its association with PTB was not proven.

The study by Bustos et al. [32] investigated the association between elected SNPs of *PGR* and PTB. It also tested whether the association between plasma concentrations of 17-alpha-hydroxyprogesterone caproate (17OHP-C) and PTB varied by elected SNPs of *PGR*. On the other hand, Manuck et al. [12] primarily investigated the responsiveness of mothers with PTB or spontaneous miscarriage in personal anamnesis on therapy with 17OHP-C concerning mothers’ carriers of elected SNPs of *PGR.* Both of these studies included just mothers in the study groups, one with and the other without PTB or spontaneous miscarriage in personal anamnesis, and both investigations included study subjects of different races, such as African American, Caucasian, and others.

While these two abovementioned studies included just mothers in their research, Ehn et al. and Mann et al. included, just like our study, mother–newborn pairs [29], or even whole family triads, including newborns, mothers and fathers, and mothers’ parents. Also, it is important to say that Ehn et al. included twin pregnancies in their research [31]. Mann et al. did not find an association of either mothers’ or newborns’ rs653752 with a higher or lower possibility of PTB. On the other hand, Ehn et al. found an association of mothers with rs653752 with PTB in a group of all singleton pregnancies, and an association of mothers with rs653752 with the middle gestation age group of PTB.

In their research, Ehn et al. also found that rs653752 was in complete linkage disequilibrium with the PROGINS variant of *PGR*, and it is hypothesized that this variant of *PGR*, like the PROGINS variant, encodes PR; as a result, PR becomes slightly less sensitive to the influence of P4 [29]. So far, a possible association of this SNP of *PGR* has only been investigated in breast cancer [46] and PTB [29]. As we have already mentioned, in our previous study, we showed that the rs1042838 and rs1042839 SNPs of *PGR* have a statistically significant association with PTB [36]. Now, we show that the rs653752 SNP of *PGR* is correlated with PTB too. Considering the location and the similar effects of rs1042838, rs1042839, and rs653752 SNPs on the sensitivity of PRs to P4 and their similar influence on the expression of *PGR*, perhaps it is not surprising that rs653752 stood out, as the first two did, in terms of the results and statistical significance in connection with a PTB. Ehn and colleagues showed in their study that these three SNPs are in allelic linkage disequilibrium [29], which does not necessarily mean all three polymorphisms have the same function but may indicate the possibility of the common inheritance of their effect.

P4 and its receptor play a crucial role in maintaining pregnancy by promoting uterine quiescence, inhibiting inflammatory responses, and preventing cervical ripening [47]. Variants in *PGR*, such as rs653752, may alter receptor activity, expression, or downstream signaling pathways that influence these processes. Although the precise mechanism by which the CC genotype contributes to term birth remains unclear, it is possible that this variant enhances receptor binding affinity or stabilizes receptor expression, leading to a more effective progesterone response. This could, in turn, reinforce uterine relaxation and reduce the risk of PTB. Previous studies have also demonstrated the importance of progesterone supplementation in preventing PTB, particularly in high-risk populations [11]. The association between the CC genotype and term birth raises intriguing questions about individualized approaches to PTB prevention. Women with different rs653752 genotypes may respond differently to exogenous progesterone therapy, suggesting a potential avenue for personalized medicine.

On the other hand, the SNP rs4754732 of *PGR* in our study did not show a statistically significant difference between groups of mothers with PTB and those with term births or between premature and at-term newborns. This finding contrasts with previous studies that have explored the role of this polymorphism in PTB. Notably, Ehn et al. reported a significant association between rs4754732 and PTB across singleton pregnancies, whereas Manuck et al. did not find such an association across different racial and PTB groups [12,29]. Furthermore, beyond pregnancy-related outcomes, rs4754732 has also been implicated in breast cancer, highlighting its broader significance in hormonal regulation and progesterone receptor function [48].

Located in the promoter region of the *PGR* gene, rs4754732 may influence the balance between progesterone receptor isoforms PR-A and PR-B. This ratio is crucial for proper progesterone signaling, as PR-A primarily modulates progesterone’s anti-inflammatory and uterine quiescence effects, while PR-B enhances progesterone responsiveness in reproductive tissues [47]. Given that progesterone plays a key role in maintaining pregnancy by preventing uterine contractions and cervical ripening, any genetic variation that alters PGR expression or function could potentially contribute to PTB risk.

A possible explanation for the lack of significant findings in our study could be the interplay between genetic, epigenetic, and environmental factors. Previous research suggests that the association between rs4754732 and PTB may be influenced by fetal sex, with one study reporting a statistically significant effect only when mothers carried male newborns [49]. Additionally, other maternal and fetal characteristics, such as maternal age, body mass index, smoking habits, and history of vaginal bleeding, are known to interact with genetic predispositions to influence PTB risk [50,51]. The variability in findings across studies suggests that rs4754732 alone may not be a strong predictor of PTB but could contribute to a more complex network of risk factors that require further investigation. Future research should focus on large-scale, multi-ethnic studies to clarify the role of rs4754732 in pregnancy outcomes. Functional studies examining its impact on *PGR* expression and progesterone signaling pathways could also provide valuable insights into its biological significance. Additionally, investigating how this SNP interacts with environmental and hormonal factors, including progesterone supplementation therapy, could further elucidate its role in pregnancy maintenance and preterm birth susceptibility [11].

Furthermore, the frequency of haplotypes for the two SNPs of *PGR* was examined. A haplotype is a set of alleles located at different locations on the same chromosome and inherited together [52]. The determination of haplotypes allows the analysis of the joint effect of multiple alleles at different loci on the same chromosome, which often do not come to the fore when these loci are targeted [52]. In this study, combinations of SNPs in the sequences of the two SNPs were investigated. The association of the T G haplotype of SNPs rs4754732–rs653752 of *PGR* was observed in the group of mothers with a higher incidence of PTBs (*p* = 0.08, Table 6). Mothers with this haplotype have a 1.5 times higher chance of PTB. The observed trend suggests that the combined effect of these SNPs may influence pregnancy outcomes. Given that rs4754732 is located in the promoter region of *PGR* and may affect the PR-A/PR-B ratio, and rs653752 is associated with receptor function, their combined impact could alter progesterone signaling pathways, potentially contributing to an increased susceptibility to PTB.

To date, there have been no studies that have included this combination of haplotypes as a possible predictor of a PTB. The study by Ehn et al. [29] was the only one that examined, among other things, the frequency of certain haplotypes in the PTB and control groups and their possible association with a PTB. The highest odds ratio for a PTB was seen in cases where four SNPs were linked in one haplotype, and in these cases, the increased odds ratio for a PTB was particularly emphasized in the case of the haplotype rs1042838–rs1042839–rs578029–rs666553. Other studies that included the SNPs mentioned did not include haplotype examination.

Given the significant association observed in our study, further large-scale investigations are needed to validate the role of the T G haplotype of SNPs rs4754732–rs653752 in PTB risk. Future research should incorporate functional studies to determine how these SNPs interact to modulate *PGR* expression and progesterone activity.

The strength of the conducted study is the strict inclusion and exclusion criteria for the investigated group of mothers with a PTB. In addition, the control group was well matched to the patient group in terms of age, parity, socioeconomic and demographic status, place of residence, antenatal care, and delivery in the same year as the patients with PTBs. An important advantage of this study is that all respondents belonged to the European population. Another advantage of this study is that both mothers and newborns were included in the study.

The main limitation of this study is the relatively small number of participants. In addition, this study did not include the study of epigenetic mechanisms that modify the DNA structure without affecting the DNA sequence, such as DNA-methylation and chromatin remodeling and changes in miRNA expression [52], which governs gene expression by balancing between the genetic and environmental factors associated with a PTB [53].

Although we included both the mothers and the newborns in the form of a mother–fetus pair in this study, which completed the complex story of the genetic link of the participants to preterm birth, an even better option would be to include a reproductive partner and expand the research to a larger sample to look more comprehensively at the complex issue of genetic predisposition to PTB.

## 4. Materials and Methods

### 4.1. Study Subjects

This case–control study was conducted between November 2017 and March 2024 at the Clinic of Gynecology and Obstetrics of the Clinical Hospital Center, Osijek, and the Department of Medical Biology and Genetics of the Faculty of Medicine, Osijek, Croatia.

Two groups of pregnant women (199 women with premature birth and 201 women with a term birth) and two groups of newborns (201 term newborns and 199 preterm newborns) participated in this study. None of the mothers in either group were genetically related.

The inclusion criterion for pregnant women in the PTB group was a live birth before 37 weeks gestation with spontaneous onset of labor and hospital admission after onset of labor. The inclusion criteria for premature-born newborns were a singleton pregnancy and the preterm birth of a liveborn infant.

The exclusion criteria for the group of preterm births were already known risk factors for preterm birth (such as in vitro fertilization, multiple pregnancies, any type of cervical surgery, inflammation of the reproductive organs, and kidney disease) as well as pregnancy complications (gestational diabetes mellitus, high blood pressure) or signs of infection in laboratory tests shortly before birth or signs of infection in the pathohistological analysis of the placenta. In addition, all newborns with congenital anomalies, proven infections, or stillbirths were excluded from the study.

The control group included healthy women who gave birth between 37 and 41 + 3 weeks of gestation. If the delivery was without complications, it ended naturally. The control group was matched with the proband women based on age, socioeconomic and demographic status, and type of prenatal care. In addition, all pregnant women who had a term birth were excluded from the study if they had a positive personal or family history of preterm birth.

The gestational age of each subject was determined according to the first day of the last menstrual cycle and was confirmed by ultrasound findings in the first trimester. In the case of a mismatch between the due date concerning the first day of the last menstrual cycle and the ultrasound finding, a correction to gestational age was made based on the ultrasound finding [54].

Sociodemographic, epidemiologic, and clinical data were collected in collaboration with the mothers. The available medical records on the pregnancy and delivery of mothers were used. Data were collected on the physical condition of the mothers during pregnancy, the family and personal history of the mothers, their habits, and previous events during pregnancy.

### 4.2. Blood Sampling and Analysis

Venous blood samples were taken from pregnant women and blood from the umbilical cord of the newborns once after obtaining informed consent. Two described genetic variants of *PGR* (rs4754732 and rs653752) were analyzed. The variants were selected based on previously published associations with a PTB [12,29,31,32]; these are listed, along with their known functions, in Table 8.

The blood was taken only once: blood was taken from the mothers after admission to the delivery room, in the first stage of labor; the blood of the newborns was taken from the umbilical cord vein immediately after birth. The genomic DNA was extracted from 200 µL EDTA-anticoagulated whole blood using commercially available spin colons for DNA extraction (QIAamp DNA Blood Mini Kit, Qiagen GmBH, Hilden, Germany) according to the manufacturer’s instructions [55]. The DNA samples were stored at −20 °C for further analysis.

SNP genotype analysis was performed using TaqMan-based fluorescent probes (Taq-Man SNP Genotyping Assays, Applied Biosystems, Foster City, CA, USA) [56] on the ABI PRISM 7500 Real-time PCR system (Applied Biosystems, Foster City, CA, USA). The thermocycling procedure consisted of the following: 1 hold at 95 °C for 10 min; 40 cycles of denaturation at 92 °C for 15 s; and primer annealing and extension at 60 °C for 1 min. Negative and positive control samples were run simultaneously within each analyzed real-time PCR plate. The total reaction volume per well was 25 µL with 2 µL of DNA used as a template. The allelic discrimination analysis was performed using SDS 7500 Software Version 2.3 (Applied Biosystems, Foster City, CA, USA).

### 4.3. Statistical Analysis

To observe the medium effect (0.25) in the differences of continuous variables, with a significance level of 0.05 and power of 0.8, the minimum required sample size was 180 patients (G*Power ver. 3.1.2).

Categorical data were presented as absolute and relative frequencies. Differences in categorical variables were tested using the Chi-square test and Fisher’s exact test. The normality of the distribution was assessed using the Shapiro–Wilk test. Continuous data were described by medians and interquartile range boundaries. Differences in continuous variables between two independent groups were analyzed using the Mann–Whitney U test (Hodges–Lehmann median difference). For all outcomes, the odds ratio (OR) and corresponding 95% confidence interval (CI) were calculated.

An additional level of quality control for genotyping was performed using the Chi-square goodness-of-fit test to compare our genotype distribution with those predicted by the Hardy–Weinberg equilibrium. Logistic regression analysis (adjusted for age, child’s gender, bleeding, and smoking) was performed to evaluate the prediction of the probability of a preterm birth. Bonferroni correction was applied for all multiple testing. All *p*-values were two-tailed, with the significance level set at alpha = 0.05. Analyses were conducted using the SNPStats web tool (Solé et al., 2006) [57], MedCalc^®^ Statistical Software version 23.0.6 (MedCalc Software Ltd., Ostend, Belgium; https://www.medcalc.org; 2024), and SPSS (IBM Corp. Released 2015. IBM SPSS Statistics for Windows, Version 23.0. Armonk, IBM Corp., New York, NY, USA).

## 5. Conclusions

The results of our study showed that there was no statistically significant difference in the distribution of genotypes and allele frequencies between groups of mothers with extremely early and early PTB compared to the group of mothers with term births. There was no statistically significant difference in the distribution of genotypes and allele frequencies between the groups of prematurely born newborns and newborns at term for either SNP of *PGR*. This research showed the association of an SNP of *PGR* (rs653752) in mothers with modulation of the risk of PTB in general and of late PTB in particular. It has an association with PTB as an SNP alone and as part of the haplotype. Namely, we found a potential association between the C allele of rs653752 in the mothers and a higher probability of term birth, as well as the association of the CC genotype of rs653752 in the mothers in the recessive inheritance model with a lower probability of PTB in general and of late PTB. We also found that the mothers who were carriers of the haplotype T-G combination of rs4754732 and rs653752 were 1.5 times more likely to have PTB, even after correcting the *p*-value for multiple comparisons. Further research on these and other PGR SNPs will certainly be needed, with the inclusion of a larger number of subjects to confirm these results. Also, the association between the CC genotype of rs653752 and reduced PTB risk highlights the potential impact of genetic variation in PGR on pregnancy outcomes. These findings emphasize the need for further investigation into genotype-specific effects on progesterone signaling and their clinical relevance for PTB prevention strategies.

## Figures and Tables

**Table 1:** Characteristics of mothers and of newborns born at term or prematurely.

	Term Birth (n = 201)	Premature Birth (n = 199)	*p* *
Mothers’ age [years] [Median (IQR)]	30 (26–34)	31 (27–35)	0.22 ^§^
Weeks of gestation [Median (IQR)]	39 + 3 (39 + 0–40 + 4)	34 + 5 (32 + 2–36 + 0)	**<0.001** ^§^
BMI [kg/m^2^] [median (IQR)]	27.4 (24.5–30.6)	26.7 (24.2–30.1)	0.28 ^§^
Number of births [median (IQR)]	2 (1–2)	1 (1–2)	0.25 ^§^
Number of PTBs (n = 30) [median (IQR)]	-	1 (1–1)	-
Number of PTBs in family history (n = 32) [median (IQR)]	-	1 (1–1)	-
Birth weight [grams] [median (IQR)]	3455 (3130–3800)	2430 (1819.35–2780.0)	**<0.001** ^§^
Mothers’ age [n (%)]			
Less than 35	158 (79)	138 (69)	**0.04**
More than 35	43 (21)	61 (31)	
Newborns’ gender [n (%)]			
Male	100 (49.8)	115 (57.8)	0.11
Female	101 (50.2)	84 (42.2)	
PTB in personal anamnesis [n (%)]	1 (0.5)	29 (15)	**<0.001**
PTB in family anamnesis [n (%)]	0	32 (16)	**<0.001**
Coffee consumption [n (%)]	168 (84)	156 (78)	0.20 ^†^
Smoking habit [n (%)]	52 (26)	63 (32)	0.20
Complications in pregnancy [n (%)]	104 (52)	151 (76)	**<0.001**
Uroinfection	18 (9)	26 (13)	0.19
Positive cervical swab	43 (22)	34 (17)	0.28
Vaginal bleeding during pregnancy [n (%)]	14 (7)	46 (23)	**<0.001**
PPROM [n (%)]	47 (23)	118 (59)	**<0.001**

Bold denotes statistical significance. Abbreviations: IQR: interquartile range; PTB: premature birth; PPROM: preterm premature rupture of membranes; * Chi-square test; ^†^ Fisher’s exact test; ^§^ Mann–Whitney U test.

**Table 2:** Genotype distribution and allele frequencies of two selected SNPs of *PGR* in mothers and newborns with premature birth and the respective controls.

		Genotype [n (%)] Mothers		OR (95% CI)	*p* *		Genotype [n (%)] Newborns		OR (95% CI)	*p* *
		Controls (n = 201)	PTB (n = 199)				Controls (n = 201)	PTB (n = 199)		
^a^ rs4754732	TT	102 (50.8)	103 (51.8)	1	0.69	TT	97 (48.5)	102 (51.3)	1	0.28
Genotype	CT	82 (40.8)	75 (37.7)	0.91 (0.60–1.37)		CT	89 (44.5)	76 (38.2)	0.81 (0.54–1.23)	
	CC	17 (8.5)	21 (10.6)	1.22 (0.61–2.45)		CC	14 (7)	21 (10.6)	1.43 (0.69–2.96)	
Allele	T	286 (71)	281 (71)	0.97 (0.72–1.32)	0.87	T	283 (71)	280 (70)	0.98 (0.72–1.33)	0.90
	C	116 (29)	117 (29)			C	117 (29)	118 (30)		
^b^ rs653752	GG	71 (35.3)	82 (41.2)	1	0.06	GG	68 (33.8)	75 (37.9)	1	0.68
Genotype	CG	86 (42.8)	91 (45.7)	0.92 (0.59–1.41)		CG	99 (49.2)	93 (47)	0.85 (0.55–1.31)	
	CC	44 (21.9)	26 (13.1)	0.51 (0.29–0.91)		CC	34 (16.9)	30 (15.2)	0.80 (0.44–1.44)	
Allele	G	228 (57)	255 (64)	**1.36 (1.02–1.81)**	**0.03**	G	235 (58)	243 (61)	1.13 (0.85–1.50)	0.40
	C	174 (43)	143 (36)			C	167 (42)	153 (39)		

^a^—Mothers: Hardy–Weinberg equilibrium: premature birth *p* = 0.23; control *p* > 0.99; newborns: premature birth *p* = 0.24; control *p* = 0.39. ^b^—Mothers: Hardy–Weinberg equilibrium: premature birth *p* > 0.99; control *p* = 0.08; newborns: premature birth *p* = 0.88; control *p* = 0.89. * Chi-square test. Abbreviations: SNP, single-nucleotide polymorphism; *PGR*, progesterone receptor gene; OR, odds ratio; CI, confidence interval; PTB, premature birth; bold denotes statistical significance.

**Table 3:** Models of inheritance of two investigated SNPs of *PGR* and their distribution between the groups of mothers with preterm and term births.

		Genotype [n (%)] Mothers		OR (95% CI)	*p* *		Genotype [n (%)] Newborns		OR (95% CI)	*p* *
		Controls (n = 201)	PTB (n = 199)				Controls (n = 201)	PTB (n = 199)		
rs4754732	TT	102 (50.8)	103 (51.8)	1	0.84	TT	97 (48.5)	102 (51.3)	1	0.58
Dominant	CT/CC	99 (49.2)	96 (48.2)	0.96 (0.65–1.42)		CT/CC	103 (51.5)	97 (48.7)	0.90 (0.60–1.33)	
Recessive	T/T-C/T	184 (91.5)	178 (89.5)	1	0.47	T/T-C/T	186 (93)	178 (89.5)	1	0.21
	C/C	17 (8.5)	21 (10.6)	1.28 (0.65–2.50)		C/C	14 (7)	21 (10.6)	1.57 (0.77–3.18)	
Overdominant	T/T-C/C	119 (59.2)	124 (62.3)	1	0.52	T/T-C/C	111 (55.5)	123 (61.8)	1	0.20
	C/T	82 (40.8)	75 (37.7)	0.88 (0.59–1.31)		C/T	89 (44.5)	76 (38.2)	0.77 (0.52–1.15)	
rs653752	GG	71 (35.3)	82 (41.2)	1	0.23	GG	68 (33.8)	75 (37.9)	1	0.40
Dominant	CC/CG	130 (64.7)	117 (58.8)	0.78 (0.52–1.17)		CC/CG	133 (66.2)	123 (62.1)	0.84 (0.56–1.26)	
Recessive	G/G-C/G	157 (78.1)	173 (86.9)	1	**0.02**	G/G-C/G	167 (83.1)	168 (84.8)	1	0.63
	C/C	44 (21.9)	26 (13.1)	**0.54 (0.32–0.91)**		C/C	34 (16.9)	30 (15.2)	0.88 (0.51–1.50)	
Overdominant	G/G-C/C	115 (57.2)	108 (54.3)	1	0.55	G/G-C/C	102 (50.8)	105 (53)	1	0.65
	C/G	86 (42.8)	91 (45.7)	1.13 (0.76–1.67)		C/G	99 (49.2)	93 (47)	0.91 (0.62–1.35)	

* Chi-square test. Abbreviations: PTB, premature birth; SNP, single-nucleotide polymorphism; *PGR*, progesterone receptor gene; OR, odds ratio; CI, confidence interval; bold denotes statistical significance.

**Table 4:** Genotype distribution and allele frequencies of two selected SNPs of *PGR* in mothers and newborns with a late premature birth and the respective controls.

		Genotype [n (%)] Mothers		OR (95 % CI)	*p* *		Genotype [n (%)] Newborns		OR (95 % CI)	*p* *
		Control (n = 201)	Late PTB (n = 150)				Control (n = 201)	Late PTB (n = 150)		
^a^ rs4754732	TT	102 (50.8)	74 (49.3)	1	0.88	TT	97 (48.5)	77 (51.3)	1	0.31
	CT	82 (40.8)	61 (40.7)	1.03 (0.66–1.60)		CT	89 (44.5)	57 (38)	0.81 (0.52–1.26)	
	CC	17 (8.5)	15 (10)	1.22 (0.57–2.59)		CC	14 (7)	16 (10.7)	1.44 (0.66–3.13)	
Allele	T	286 (71)	209 (70)	0.93 (0.67–1.29)	0.67	T	283 (71)	211 (70)	0.98 (0.71–1.36)	0.90
	C	116 (29)	91 (30)			C	117 (29)	89 (30)		
^b^ rs653752	GG	71 (35.3)	62 (41.3)	1	**0.02**	GG	68 (33.8)	61 (40.9)	1	0.39
	CG	86 (42.8)	72 (48)	0.96 (0.60–1.52)		CG	99 (49.2)	65 (43.6)	0.73 (0.46–1.17)	
	CC	44 (21.9)	16 (10.7)	**0.42 (0.21–0.81)**		CC	34 (16.9)	23 (15.4)	0.75 (0.40–1.42)	
Allele	G	228 (57)	196 (65)	**1.44 (1.06–1.96)**	**0.02**	G	235 (58)	187 (63)	1.20 (0.88–1.63)	0.25
	C	174 (43)	104 (35)			C	167 (42)	111 (37)		

^a^—Mothers: Hardy–Weinberg equilibrium: premature birth *p* = 0.49; control *p* = 0.41; newborns: premature birth *p* = 0.33; control *p* = 0.39. ^b^—Mothers: Hardy–Weinberg equilibrium: premature birth *p* = 0.41; control *p* = 0.48; newborns: premature birth *p* = 0.48; control *p* = 0.89. * Chi-square test. Abbreviations: SNP, single-nucleotide polymorphism; *PGR*, progesterone receptor gene; OR, odds ratio; CI, confidence interval; PTB: premature birth; bold denotes statistical significance.

**Table 5:** Models of inheritance of the two investigated SNPs of *PGR* and their distribution between the groups of mothers with late preterm and term births.

		Genotype [n (%)] Mothers		OR (95% CI)	*p* *		Genotype [n (%)] Newborns		OR (95% CI)	*p* *
		Control (n = 201)	Late PTB (n = 150)				Control (n = 201)	Late PTB (n = 150)		
rs4754732	TT	102 (50.8)	74 (49.3)	1	0.79	TT	97 (48.5)	77 (51.3)	1	0.60
Dominant	CT/CC	99 (49.2)	76 (50.7)	1.06 (0.69–1.62)		CT/CC	103 (51.5)	73 (48.7)	0.89 (0.58–1.36)	
Recessive	T/T-C/T	184 (91.5)	135 (90)	1	0.62	T/T-C/T	186 (93)	134 (89.3)	1	0.23
	C/C	17 (8.5)	15 (10)	1.20 (0.58–2.49)		C/C	14 (7)	16 (10.7)	1.59 (0.75–3.36)	
Overdominant	T/T-C/C	119 (59.2)	89 (59.3)	1	0.98	T/T-C/C	111 (55.5)	93 (62)	1	0.22
	C/T	82 (40.8)	61 (40.7)	0.99 (0.65–1.53)		C/T	89 (44.5)	57 (38)	0.76 (0.50–1.18)	
rs653752	GG	71 (35.3)	62 (41.3)	1	0.25	GG	68 (33.8)	61 (40.9)	1	0.17
Dominant	CC/CG	130 (64.7)	88 (58.7)	0.78 (0.50–1.20)		CC/CG	133 (66.2)	88 (59.1)	0.74 (0.48–1.14)	
Recessive	G/G-C/G	157 (78.1)	134 (89.3)	1	**0.005**	G/G-C/G	167 (83.1)	126 (84.6)	1	0.71
	C/C	44 (21.9)	16 (10.7)	**0.43 (0.23–0.79)**		C/C	34 (16.9)	23 (15.4)	0.90 (0.50–1.60)	
Overdominant	G/G-C/C	115 (57.2)	78 (52)	1	0.33	G/G-C/C	102 (50.8)	84 (56.4)	1	0.30
	C/G	86 (42.8)	72 (48)	1.23 (0.81–1.89)		C/G	99 (49.2)	65 (43.6)	0.80 (0.52–1.22)	

* Chi-square test. Abbreviations: PTB, premature birth; SNP, single-nucleotide polymorphisms; *PGR*, progesterone receptor gene; OR, odds ratio; CI, confidence interval; bold denotes statistical significance.

**Table 6:** Frequency of haplotypes of the two SNPs of *PGR* and their impact on the predisposition to a PTB.

Mothers	Haplotype		Controls (%)	PTB (%)	OR (95% CI)	*p* * ^†^
	rs4754732	rs653752				
1	T	C	41.9	33.5	1.0	-
2	T	G	29.2	37.2	**1.59 (1.13–2.24)**	**0.008**
3	C	G	27.5	26.9	1.20 (0.85–1.68)	0.30
4	C	C	1.4	2.5	2.15 (0.65–7.19)	0.21
**Newborns**	**Haplotype**		**Controls (%)**	**PP (%)**	**OR (95% CI)**	*p* * **^†^**
	**rs4754732**	**rs653752**				
1	T	C	40.8	37.4	1.0	-
2	T	G	29.9	32.9	01.20 (0.86–1.67)	0.29
3	C	G	28.5	28.6	1.10 (0.78–1.55)	0.60
RARE	*	*	0.8	1.1	1.58 (0.25–9.90)	0.63

* χ^2^ test; ^†^ Bonferroni correction. Abbreviations: SNP, single-nucleotide polymorphism; *PGR*, progesterone receptor gene; PTB, premature birth; OR, odds ratio; CI, confidence interval; bold denotes statistical significance.

**Table 7:** Prediction of the probability of a preterm birth (bivariate logistic regression) (adjusted for age, child’s gender, bleeding, and smoking).

Bivariate Regression	ß	Wald	*p*	OR (95% CI)
Mothers				
rs4754732 (C/T-C/C vs. TT)	−0.05	0.05	0.82	0.95 (0.64–1.43)
rs653752 (C/G-C/C vs. GG)	−0.26	1.46	0.23	0.77 (0.51–1.17)
Newborns				
rs4754732 (C/T-C/C vs. TT)	−0.04	0.04	0.85	0.96 (0.64–1.45)
rs653752 (C/G-C/C vs. GG)	−0.17	0.59	0.44	0.85 (0.55–1.29)

ß—Regression coefficient. Abbreviations: OR, odds ratio; CI, confidence interval.

**Table 8:** The studied progesterone receptor single-nucleotide polymorphisms (SNPs).

SNP	Location	Gene Region	Base Change	Citations	Related Phenotype
rs4754732	chr11:101,137,771 (GRCh38.p14)	PGR-AS1: intron region	T/A>T/C	Ehn et al., [29] Manuck et al., [12]	Premature birth
rs653752	chr11:101,077,379 (GRCh38.p14)	Intron region	C>G	Ehn et al., [29] Bustos et al., [32] Mann et al., [31] Manuck et al., [12]	Premature birth

## Data Availability

The data presented in this study are available on request from the corresponding author due to legal and ethical restrictions.

## Supplementary Materials

The following supporting information can be downloaded at: https://www.mdpi.com/article/10.3390/ijms26041606/s1.
