# Supplementary material for: Variants of the Progesterone Receptor Gene as Modulators of Risk for Idiopathic Spontaneous Premature Birth
Source: Int J Mol Sci. 2025 Feb 13;26(4):1606. doi: 10.3390/ijms26041606 (PMC11855569; doi:10.3390/ijms26041606)
Supplement: Supplementary file 1 [file ijms-26-01606-s001.zip › ijms-3436102-supplementary.pdf]

Table S1. Genotype distribution and allele frequencies of two selected SNPs of *PGR* in groups of newborns with premature birth and the respective controls

			Genotype (n (%)] NEWBORNS		Odds ratio (95 % confidence interval)	*P**
			Term birth (n = 201)	Premature birth (n = 199)		
[a]*rs4754732*		TT	97 (48.5)	102 (51.3)	1	
		CT	89 (44.5)	76 (38.2)	0.81 (0.54-1.23)	0.28
		CC	14 (7)	21 (10.6)	1.43 (0.69-2.96)	
	Allele	T	283 (71)	280 (70)	0.98 (0.72 – 1.33)	0.90
		C	117 (29)	118 (30)		
[b]*rs653752*		GG	68 (33.8)	75 (37.9)	1	
		CG	99 (49.2)	93 (47)	0.85 (0.55-1.31)	0.68
		CC	34 (16.9)	30 (15.2)	0.80 (0.44-1.44)	
	Allele	G	235 (58)	243 (61)	1.13 (0.85 – 1.50)	0.40
		C	167 (42)	153 (39)		

a- Hardy-Weinberg equilibrium: premature birth P = 0.24; term birth P = 0.39

b - Hardy-Weinberg equilibrium: premature birth = 0.88; term birth P = 0.89

*Chi-square test. Abbreviations: SNP, single-nucleotide polymorphism; *PGR* – progesterone receptor gene

Table S2. Models of inheritance of two investigated SNPs of *PGR* and their distribution in groups of newborns with premature birth and the respective controls

		Genotype [n (%)] NEWBORNS		Odds ratio (95% confidence interval)	*P**
		Term birth (n = 201)	Premature birth (n = 199)		
rs4754732	TT	97 (48.5)	102 (51.3)	1	0.58
DOMINANT	CT/CC	103 (51.5)	97 (48.7)	0.90 (0.60-1.33)	
RECESSIVE	T/T-C/T	186 (93)	178 (89.5)	1	0.21
	C/C	14 (7)	21 (10.6)	1.57 (0.77-3.18)	
SUPERDOMINAT	T/T-C/C	111 (55.5)	123 (61.8)	1	0.20
	C/T	89 (44.5)	76 (38.2)	0.77 (0.52-1.15)	
rs653752	GG	68 (33.8)	75 (37.9)	1	0.40
DOMINANT	CC/CG	133 (66.2)	123 (62.1)	0.84 (0.56-1.26)	
RECESSIVE	G/G-C/G	167 (83.1)	168 (84.8)	1	0.63
	C/C	34 (16.9)	30 (15.2)	0.88 (0.51-1.50)	
SUPERDOMINANT	G/G-C/C	102 (50.8)	105 (53)	1	0.65
	C/G	99 (49.2)	93 (47)	0.91 (0.62-1.35)	

*Chi-square test. Abbreviations: SNP, single-nucleotide polymorphism; *PGR* – progesterone receptor gene

Table S3. Genotype distribution and allele frequencies of two selected SNPs of *PGR* in groups of mothers with extremely early premature birth and the respective controls

			Genotype [n (%)] MOTHERS		Odds ratio (95 % confidence interval)	*P**
			Term birth (n = 201)	Extremely early premature birth (n = 16)		
[a]rs4754732		TT	102 (50.8)	8 (50)	1	
		CT	82 (40.8)	6 (37.5)	0.93 (0.31-2.80)	0.87
		CC	17 (8.5)	2 (12.5)	1.50 (0.29-7.67)	
	Allele	T	286 (71)	22 (69)	0.89 (0.41 – 1.94)	0.77
		C	116 (29)	10 (31)		
[b]rs653752		GG	71 (35.3)	9 (56.2)	1	
		CG	86 (42.8)	4 (25)	0.37 (0.11-1.24)	0.23
		CC	44 (21.9)	3 (18.8)	0.54 (0.14-2.10)	
	Allele	G	228 (57)	22 (69)	1.68 (0.77 – 3.64)	0.19
		C	174 (43)	10 (31)		

a - Hardy-Weinberg equilibrium: premature birth P = 0.50; term birth P = 0.38

b - Hardy-Weinberg equilibrium: premature birth P = 0.11; term birth P = 0.08

*Chi-square test. Abbreviations: SNP, single-nucleotide polymorphism; *PGR* – progesterone receptor gene

Table S4. Models of inheritance of two investigated SNPs of *PGR* and their distribution in the groups of mothers with extremely early premature birth and the respective controls

		Genotype [n (%)] MOTHERS		Odds ratio (95 % confidence interval)	*P**
		Term birth (n = 201)	Extremely early premature birth (n = 16)		
rs4754732	TT	102 (50.8)	8 (50)	1	0.95
DOMINANT	CT/CC	99 (49.2)	8 (50)	1.03 (0.37-2.85)	
RECESSIVE	T/T-C/T	184 (91.5)	14 (87.5)	1	0.60
	C/C	17 (8.5)	2 (12.5)	1.55 (0.32-7.38)	
SUPERDOMINANT	T/T-C/C	119 (59.2)	10 (62.5)	1	0.80
	C/T	82 (40.8)	6 (37.5)	0.87 (0.30-2.49)	
rs653752	GG	71 (35.3)	9 (56.2)	1	0.10
DOMINANT	CC/CG	130 (64.7)	7 (43.8)	0.42 (0.15-1.19)	
RECESSIVE	G/G-C/G	157 (78.1)	13 (81.2)	1	0.77
	C/C	44 (21.9)	3 (18.8)	0.82 (0.22-3.02)	
SUPERDOMINANT	G/G-C/C	115 (57.2)	12 (75)	1	0.15
	C/G	86 (42.8)	4 (25)	0.45 (0.14-1.43)	

*Chi-square test. Abbreviations: SNP, single-nucleotide polymorphism; *PGR* – progesterone receptor gene

Table S5. Genotype distribution and allele frequencies of two selected SNPs of *PGR* in groups of newborns with extremely early premature birth and the respective controls

			Genotype [n (%)] NEWBORNS		Odds ratio (95 % confidence interval)	*P**
			Term birth (n = 201)	Extremely early premature birth (n = 16)		
[a]rs4754732		TT	97 (48.5)	10 (62.5)	1	0.28
		CT	89 (44.5)	4 (25)	0.44 (0.13-1.44)	
		CC	14 (7)	2 (12.5)	1.39 (0.27-6.99)	
	Allele	T	283 (71)	24 (75)	1.24 (0.54 – 2.84)	0.61
		C	117 (29)	8 (25)		
[b]rs653752		GG	97 (48.5)	10 (62.5)	1	0.88
		CG	89 (44.5)	4 (25)	0.44 (0.13-1.44)	
		CC	14 (7)	2 (12.5)	1.39 (0.27-6.99)	
	Allele	G	235 (58)	20 (62)	1.18 (0.56 – 2.49)	0.66
		C	167 (42)	12 (38)		

a - Hardy-Weinberg equilibrium: premature birth = 0.20; term birth P = 0.39

b - Hardy-Weinberg equilibrium: premature birth P > 0.99; term birth P = 0.89

*Chi-square test. Abbreviations: SNP, single-nucleotide polymorphism; *PGR* – progesterone receptor gene

Table S6. Models of inheritance of two investigated SNPs of *PGR* and their distribution in the groups of newborns with extremely early premature birth and the respective controls

		Genotype [n (%)] NEWBORNS		Odds ratio (95 % confidence interval)	*P**
		Term birth (n = 201)	Extremely early premature birth (n = 16)		
rs4754732	TT	97 (48.5)	10 (62.5)	1	0.28
DOMINANT	CT/CC	103 (51.5)	6 (37.5)	0.57 (0.20-1.61)	
RECESSIVE	T/T-C/T	186 (93)	14 (87.5)	1	0.46
	C/C	14 (7)	2 (12.5)	1.90 (0.39-9.20)	
SUPERDOMINANT	T/T-C/C	111 (55.5)	12 (75)	1	0.12
	C/T	89 (44.5)	4 (25)	0.42 (0.13-1.33)	
rs653752	GG	68 (33.8)	6 (37.5)	1	0.77
DOMINANT	CC/CG	133 (66.2)	10 (62.5)	0.85 (0.30-2.44)	
RECESSIVE	G/G-C/G	167 (83.1)	14 (87.5)	1	0.64
	C/C	34 (16.9)	2 (12.5)	0.70 (0.15-3.23)	
SUPERDOMINANT	G/G-C/C	102 (50.8)	8 (50)	1	0.95
	C/G	99 (49.2)	8 (50)	1.03 (0.37-2.85)	

*Chi-square test. Abbreviations: SNP, single-nucleotide polymorphism; *PGR* – progesterone receptor gene

Table S7. Genotype distribution and allele frequencies of two selected SNPs of *PGR* in groups of newborns with early premature birth and the respective controls

			Genotype [n (%)] NEWBORNS		Odds ratio (95 % confidence interval)	*P**
			Term birth (n = 201)	Early premature birth (n = 33)		
[a]rs4754732		TT	97 (48.5)	15 (45.5)	1	
		CT	89 (44.5)	15 (45.5)	1.09 (0.50-2.36)	0.90
		CC	14 (7)	3 (9.1)	1.39 (0.36-5.40)	
	Allele	T	283 (71)	45 (68)	0.89 (0.51 – 1.55)	0.67
		C	117 (29)	21 (32)		
[b]rs653752		GG	68 (33.8)	8 (24.2)	1	
		CG	99 (49.2)	20 (60.6)	1.72 (0.72-4.12)	0.45
		CC	34 (16.9)	5 (15.2)	1.25 (0.38-4.11)	
	Allele	G	235 (58)	36 (55)	0.85 (0.51 – 1.44)	0.55
		C	167 (42)	30 (45)		

a - Hardy-Weinberg equilibrium: premature birth P > 0.99; term birth P = 0.39

fb- Hardy-Weinberg equilibrium: premature birth P = 0.30; term birth P = 0.89

*Chi-square test. Abbreviations: SNP, single-nucleotide polymorphism; *PGR* – progesterone receptor gene

Table S8. Models of inheritance of two investigated SNPs of *PGR* and their distribution in the groups of newborns with early premature birth and the respective controls

		Genotype [n (%)] NEWBORNS		Odds ratio (95 % confidence interval)	*P**
		Term birth (n = 201)	Early premature birth (n = 33)		
rs4754732	TT	97 (48.5)	15 (45.5)	1	0.75
DOMINANT	CT/CC	103 (51.5)	18 (54.5)	1.13 (0.54-2.37)	
RECESSIVE	T/T-C/T	186 (93)	30 (90.9)	1	0.68
	C/C	14 (7)	3 (9.1)	1.33 (0.36-4.90)	
SUPERDOMINANT	T/T-C/C	111 (55.5)	18 (54.5)	1	0.92
	C/T	89 (44.5)	15 (45.5)	1.04 (0.50-2.18)	
rs653752	GG	68 (33.8)	8 (24.2)	1	0.27
DOMINANT	CC/CG	133 (66.2)	25 (75.8)	1.60 (0.68-3.73)	
RECESSIVE	G/G-C/G	167 (83.1)	28 (84.8)	1	0.80
	C/C	34 (16.9)	5 (15.2)	0.88 (0.32-2.43)	
SUPERDOMINANT	G/G-C/C	102 (50.8)	13 (39.4)	1	0.22
	C/G	99 (49.2)	20 (60.6)	1.59 (0.75-3.36)	

*Chi-square test. Abbreviations: SNP, single-nucleotide polymorphism; *PGR* – progesterone receptor gene

Table S9. Genotype distribution and allele frequencies of two selected SNPs of *PGR* in groups of newborns with late premature birth and the respective controls

			Genotype [n (%)] NEWBORNS		Odds ratio (95 % confidence interval)	*P**
			Term birth (n = 201)	Late premature birth (n = 150)		
[a]rs4754732		TT	97 (48.5)	77 (51.3)	1	
		CT	89 (44.5)	57 (38)	0.81 (0.52-1.26)	0.31
		CC	14 (7)	16 (10.7)	1.44 (0.66-3.13)	
	Allele	T	283 (71)	211 (70)	0.98 (0.71 – 1.36)	0.90
		C	117 (29)	89 (30)		
[b]rs653752		GG	68 (33.8)	61 (40.9)	1	
		CG	99 (49.2)	65 (43.6)	0.73 (0.46-1.17)	0.39
		CC	34 (16.9)	23 (15.4)	0.75 (0.40-1.42)	
	Allele	G	235 (58)	187 (63)	1.20 (0.88 – 1.63)	0.25
		C	167 (42)	111 (37)		

a - Hardy-Weinberg equilibrium: premature birth P = 0.33; term birth P = 0.39

b- Hardy-Weinberg equilibrium: premature birth P = 0.48; term birth P = 0.89

*Chi-square test. Abbreviations: SNP, single-nucleotide polymorphism; *PGR* – progesterone receptor gene

Table S10. Models of inheritance of two investigated SNPs of *PGR* and their distribution in the groups of newborns with late premature birth and the respective controls

		Genotype [n (%)] NEWBORNS		Odds ratio (95 % confidence interval)	*P**
		Term birth (n = 201)	Late premature birth (n = 150)		
rs4754732	TT	97 (48.5)	77 (51.3)	1	0.60
DOMINANT	CT/CC	103 (51.5)	73 (48.7)	0.89 (0.58-1.36)	
RECESSIVE	T/T-C/T	186 (93)	134 (89.3)	1	0.23
	C/C	14 (7)	16 (10.7)	1.59 (0.75-3.36)	
SUPERDOMINANT	T/T-C/C	111 (55.5)	93 (62)	1	0.22
	C/T	89 (44.5)	57 (38)	0.76 (0.50-1.18)	
rs653752	GG	68 (33.8)	61 (40.9)	1	0.17
DOMINANT	CC/CG	133 (66.2)	88 (59.1)	0.74 (0.48-1.14)	
RECESSIVE	G/G-C/G	167 (83.1)	126 (84.6)	1	0.71
	C/C	34 (16.9)	23 (15.4)	0.90 (0.50-1.60)	
SUPERDOMINANT	G/G-C/C	102 (50.8)	84 (56.4)	1	0.30
	C/G	99 (49.2)	65 (43.6)	0.80 (0.52-1.22)	

*Chi-square test. Abbreviations: SNP, single-nucleotide polymorphism; *PGR* – progesterone receptor gene
